# Effects of aging on cognitive and brain inter-network integration patterns underlying usual and dual-task gait performance

**DOI:** 10.3389/fnagi.2022.956744

**Published:** 2022-09-28

**Authors:** Amgad Droby, Eleanna Varangis, Christian Habeck, Jeffrey M. Hausdorff, Yaakov Stern, Anat Mirelman, Inbal Maidan

**Affiliations:** ^1^Laboratory for Early Markers of Neurodegeneration (LEMON), Center for the Study of Movement, Cognition, and Mobility (CMCM), Tel Aviv Sourasky Medical Center, Neurological Institute, Tel Aviv, Israel; ^2^Department of Neurology, Sackler Faculty of Medicine, Tel Aviv University, Tel Aviv-Yafo, Israel; ^3^Sagol School of Neuroscience, Tel Aviv University, Tel Aviv-Yafo, Israel; ^4^Cognitive Neuroscience Division, Department of Neurology, Columbia University, New York, NY, United States; ^5^Department of Orthopedic Surgery, Rush Alzheimer’s Disease Center, Rush University, Chicago, IL, United States; ^6^Department of Physical Therapy, Sackler Faculty of Medicine, Tel Aviv University, Tel Aviv, Israel

**Keywords:** cognitive reserve, gait, inter-network connectivity, aging, functional connectivity

## Abstract

**Introduction:**

Aging affects the interplay between cognition and gait performance. Neuroimaging studies reported associations between gait performance and structural measures; however, functional connectivity (FC) analysis of imaging data can help to identify dynamic neural mechanisms underlying optimal performance. Here, we investigated the effects on divergent cognitive and inter-network FC patterns underlying gait performance during usual (UW) and dual-task (DT) walking.

**Methods:**

A total of 115 community-dwelling, healthy participants between 20 and 80 years were enrolled. All participants underwent comprehensive cognitive and gait assessments in two conditions and resting state functional MRI (fMRI) scans. Inter-network FC from motor-related to 6 primary cognitive networks were estimated. Step-wise regression models tested the relationships between gait parameters, inter-network FC, neuropsychological scores, and demographic variables. A threshold of *p* < 0.05 was adopted for all statistical analyses.

**Results:**

UW was largely associated with FC levels between motor and sustained attention networks. DT performance was associated with inter-network FC between motor and divided attention, and processing speed in the overall group. In young adults, UW was associated with inter-network FC between motor and sustained attention networks. On the other hand, DT performance was associated with cognitive performance, as well as inter-network connectivity between motor and divided attention networks (VAN and SAL). In contrast, the older age group (> 65 years) showed increased integration between motor, dorsal, and ventral attention, as well as default-mode networks, which was negatively associated with UW gait performance. Inverse associations between motor and sustained attention inter-network connectivity and DT performance were observed.

**Conclusion:**

While UW relies on inter-network FC between motor and sustained attention networks, DT performance relies on additional cognitive capacities, increased motor, and executive control network integration. FC analyses demonstrate that the decline in cognitive performance with aging leads to the reliance on additional neural resources to maintain routine walking tasks.

## Introduction

Gait is a complex behavior that involves the integration of motor, sensory and cognitive functions (Rosso et al., 2013). A decline in higher cognitive performance including executive functioning and memory was reported by several studies to be linked to gait performance decline and falls (Yogev-Seligmann et al., 2008; Montero-Odasso et al., 2012; Morris et al., 2016; Sunderaraman et al., 2019). These findings highlight the cognitive-motor interactions that take place during walking and their association with adverse health outcomes associated with aging. Dual-task (DT) walking paradigms have also been used to understand the relationship between cognition and gait. These are based on the general observation that when confronted with two attention-demanding tasks, participants may prioritize one task over the other depending on their compensatory capabilities and available cognitive and/or motor reserves (Yogev-Seligmann et al., 2008). During DT walking, deterioration in the performance of either the motor or the cognitive task when they are attempted simultaneously is defined as the DT cost (Plummer-D’Amato et al., 2008). This can manifest as alterations in walking patterns (i.e., reduced gait velocity, increased gait variability), or as decline in cognitive task performance across visuomotor processing, verbal fluency, or working memory domains (Patel et al., 2014). Gait performance predicts survival while DT walking predicts cognitive decline and the development of dementia (Montero-Odasso et al., 2017; Ramirez and Gutierrez, 2021).

Imaging studies have explored the structural and functional underpinnings of cognitive and motor performance. Gait performance changes during usual and DT walking conditions were associated with reduced gray matter (GM) volumes, and cortical thickness in several brain regions (Ezzati et al., 2015; Stijntjes et al., 2016). However, while such structural measures might be sensitive to the brain’s anatomic reserve, functional neuroimaging metrics such as functional MRI (fMRI) or resting-state (rs-) fMRI might be more sensitive in identifying active compensatory mechanisms taking place (Maidan et al., 2021). In fact, altered functional connectivity (FC) patterns within brain networks were found across several studies to be associated with gait performance in older adults (Yuan et al., 2015; Hsu et al., 2019, 2020; Maidan et al., 2019, 2020; Lammers et al., 2020). Similarly, another study by Lammers et al. (2020) investigated the relationship between the supplementary motor network FC and clinical frailty assessed using Fried’s frailty score in older adults. The authors of this study demonstrated reduced SMA FC in frail participants compared to levels measured in pre-frail and clinically preserved participants in this region is associated with clinical motor and cognitive performance (Lammers et al., 2020). In a recent study, we investigated the association between cognitive outcomes and functional brain networks at rest across the adult lifespan (Varangis et al., 2019). The results showed that higher participant age was associated with weaker within-network connectivity, less functional distinction between individual networks (system segregation), and reductions in local efficiency. Further, many of these measures of FC were related to cognitive outcomes, such that, higher levels of within- and between-network connectivity, and greater system segregation at rest were associated with better performance on neuropsychological tasks measuring processing speed, fluid reasoning, and episodic memory. These correlations suggest that connectivity measures may play a role in accounting for some of the variability in cognitive function across the adult lifespan. Furthermore, inter-network connectivity measures are believed to reflect the ongoing re-structuring mechanisms of these neural networks, or the reduction of their specialization to maintain stable functioning (Reuter-Lorenz et al., 2000). Less is known, however, about the relationship between these connectivity measures and gait performance across the lifespan.

Building upon these findings, the present study aimed to investigate the relationship between motor-cognitive internetwork connectivity and gait performance in healthy adults. Our hypothesis was that due to the increased task demand, DT walking performance relies on the successful recruitment of additional cognitive and neural reserve capacities compared to usual walking (UW) (i.e., internetwork connectivity between motor and higher cognitive brain networks). Moreover, with aging and cognitive reserve decline, maintaining optimal gait performance can be achieved via the dynamic utilization of these neural resources.

## Materials and methods

### Study participants

The present work is based on the analysis of two larger longitudinal cohort studies of adults representing the whole adult lifespan, the Reference Ability Neural Network (12) and Cognitive Reserve studies. For the present work, *N* = 115 [Age (mean ± *SD*): 60.45 ± 13.75; Range: 27–82 years, *N* = 64 females] community-dwelling, healthy participants were enrolled using established market mailing procedures to equate the recruitment procedures of young and old adults (Stern et al., 2014). Participants were enrolled in the study if they met the inclusion criteria including: (i) right-handed, (ii) English-speaking, (iii) no outstanding orthopedic, psychiatric and/or neurological disorders that can affect either gait and/or cognition, (iv) normal or corrected-to-normal vision, (v) no CNS-targeting medications. Exclusion criteria included: (i) MRI contraindications, (ii) hearing impairment, (iii) objective cognitive or functional impairment, (iv) high blood pressure, (v) current or recent non-skin neoplastic disease or melanoma (but not prostatic carcinoma), (vi) active hepatic disease or primary renal disease requiring dialysis, (vii) primary untreated endocrine diseases (Well-treated hypothyroidism was not excluded), (viii) HIV infection or other medical disorders judged by a neurologist to interfere with study, (ix) pregnancy or lactation (participation allowed 3 months after ceasing lactation), and (x) medications that target the CNS taken within the last month. Participants with psychiatric issues were excluded if they had a history of psychosis, ECT, current or recent major depressive disorder, bipolar disorder or anxiety disorder. Neurological exclusions included brain disorders such as stroke, tumor, infection, epilepsy, multiple sclerosis, degenerative diseases, head injury [loss of consciousness (LOC) > 5 min], intellectual disability, imaged cortical stroke or large subcortical lacunae or infarct or space-occupying lesion (≥2 cubic cm), and diagnosed learning disability, dyslexia, or ADHD. Eligible individuals were further screened in person, and a Mattis Dementia Rating Scale (Matteau et al., 2011) score of at least 130 was required for enrollment. This study was approved by the local Columbia University Medical Center Institutional Review Board. All included participants gave their informed written consent.

### Cognitive assessment

All enrolled participants to this study completed the following tests in a fixed order. The Wechsler Adult Intelligence Scale (WAISIII; Wechsler, 1997), Letter-Number Sequencing, American National Adult Reading Test (AMNART; Wechsler, 1997), Selective Reminding Task (SRT) immediate recall (Buschke and Fuld, 1974), WAIS-III Matrix Reasoning (Wechsler, 1997), SRT delayed recall and delayed recognition (Buschke and Fuld, 1974), WAIS-III Digit Symbol (Wechsler, 1997), Trail-Making Test versions A and B (TMT-A/B; Reitan, 1978), Controlled Word Association (C-F-L) and Category Fluency (animals; Benton et al., 1983), Stroop Color Word Test (Golden, 1975), Wechsler Test of Adult Reading (WTAR; Holdnack, 2001), WAIS-III Vocabulary (Wechsler, 1997), and WAIS-III Block Design (Wechsler, 1997). Then, the performance in these tests was then clustered into four primary cognitive domains (see Salthouse, 2009; Stern et al., 2014; Razlighi et al., 2017): Episodic Memory (all SRT outcomes), Vocabulary (WAIS Vocabulary, WTAR, AMNART), Processing Speed (WAIS Digit Symbol, Stroop Color, Stroop Color Word, TMT-A), and Fluid Reasoning (WAIS Matrix Reasoning, WAIS Block Design, TMT-B). After collection of all baseline assessment data, performance on each task was z-scored relative to the mean and standard deviation for each task in the study sample. Then, task-based z-scores were averaged for each domain. Based on this, the primary cognitive outcomes included in the present study are the domain-based z-scores for performance on tasks corresponding to the four aforementioned cognitive domains.

### Gait assessment

A lightweight sensor including a three-axis accelerometer and a three-axis gyroscope (DynaPort; McRoberts, The Hague, Netherlands) was used for gait assessment. The device was placed on the lower back of the participants to assess gait during normal and DT walking. The gait protocol included walking along a 20-meter-long corridor for 1 min under two conditions: (1) preferred, usual-walking (UW) speed, and (2) dual-tasking (DT) walk while saying words that start with the letter “A” (cognitive task), without restriction on word length or number of syllables. There were no instructions for task prioritization, and the participants were not made aware of any errors or received any feedback on their performance. As previously described, the number of responses to the cognitive task during dual task gait were recorded but errors were not [ref pretty]. The spatiotemporal gait measures were obtained from the mean values of a minimum of 30 steps included stride length, velocity, stride regularity, step regularity, and step symmetry (Tura et al., 2010; Weiss et al., 2011; Kosbar et al., 2014; Mirelman et al., 2014). Briefly, accelerometer signals were filtered using a low-pass Butterworth filter with a frequency cut-off of 3.5 Hz with a band-pass of less than 0.5 db. Turns were identified from the gyroscope signal and were cut. Only straight line walking, defined as sagittal progression walking, was analyzed (Mirelman et al., 2014). Stride time was determined by automatic identification of the time between two consecutive strikes of the same foot, detected from the trunk acceleration (Mirelman et al., 2015). Stride time variability was calculated as the magnitude of stride-to-stride fluctuations, normalized to each subject’s mean stride time (Coefficient of Variation = standard deviation/meanx100) (Mirelman et al., 2015). Most participants did not stop during both walking tasks. On the account of stopping for less than 3 s, data remained continuous. If the participant stopped and discontinued the trial, the measures were calculated up to the arrest moment.

### MRI acquisition

MRI scans were performed on the same day of cognitive and gait assessments. MR images acquired using a 3 Tesla Philips Achieva (Philips, The Netherlands) using a standard quadrature head coil. Among other structural and functional scans, the imaging protocol included a high-resolution T1-weighted Magnetization Prepared Rapid Gradient Echo (MPRAGE) image (TR = 6.5 ms, TE = 3 ms; fip angle = 8°, matrix size = 256 × 256, and FOV = 256 mm, slice thickness = 1 mm, 180 slices), and a resting-state fMRI scan [TE = 20 ms, TR = 2,000 ms, Flip angle = 72°, in-plane resolution: 112 × 112 voxels; Slice thickness = 3 mm, 37 slices, duration of the collected fMRI scans was 5 (150 volumes) or 9.5 (285 volumes) min].

### MRI processing

fMRI datasets were preprocessed using an in-house developed method as described previously in Varangis et al. (2019). The preprocessing pipeline included slice-timing and motion correction performed in FSL, calculation of frame-wise displacement, replacement for contaminated volumes (Carp, 2013), band-pass filtering using fslmaths, and residualization of the processed data with respect to FWD, root mean square difference of the BOLD signal, left and right hemisphere white matter, and lateral ventricular signals (Birn et al., 2006). Anatomical T1 image segmentation was performed using FreeSurfer, and was inspected visually for any possible inaccuracies. In order to perform the inter-network FC analyses, the coordinates of the 264 ROIs identified by Power et al. (2011) were transferred to native space via non-linear registration of the subject’s structural scan to the MNI template using the ANTS software package. Next, a 10 mm radius spherical mask was generated for each coordinate and intersected with the FreeSurfer GM mask to derive the GM-registered ROI masks for each of the 264 ROIs. An intermodal, intra-subject, rigid-body registration of the fMRI reference image and T1 scan was then performed using FLIRT to fMRI space. These transferred ROI masks were used to average all voxels within each mask to obtain a single fMRI time-series for each of the 264 ROIs.

Time-series from each ROI were used to generate correlation matrices among all ROIs, and were then z-transformed to generate normalized correlation matrices for each participant. The following networks were the focus of the present study: somato-motor Hand (Hand; 30 ROIs), Default Mode (DMN; 58 ROIs), Salience (SAL; 18 ROIs), Cingulo-Opercular (CO; 14 ROIs), Fronto-parietal (FP; 25 ROIs), Dorsal Attention (DAN; 11 ROIs), Cerebellar (Cer; 4 ROIs), and Ventral Attention (VAN; 9 ROIs) (see Supplementary Material and Varangis et al., 2019 for further details and graphic illustration of the spatial distribution of these investigated networks of interest). Inter-network connectivity was estimated from the Hand and Cerebellar networks to the six primary cognitive networks (DMN, SAL, CO, FP, DAN, VAN) by computing the average correlation from the Hand and Cerebellar networks to each of the cognitive networks. Thus, there were 12 primary connectivity measures included in the present study: Hand-DMN, Hand-SAL, Hand-CO, Hand-FP, Hand-DAN, Hand-VAN, Cer-DMN, Cer-SAL, Cer-CO, Cer-FP, Cer-DAN, and Cer-VAN.

### Statistical analyses

All statistical analyses were performed using the Statistical Package for Social Science (SPSS^®^ 27, IBM) software. A step-wise forward-selection regression model tested the relationship between UW and DT gait parameters, inter-network FC *z*-scores, neuropsychological performance scores in four domains (speed, working memory, vocabulary, and reasoning), age and other demographic variables (age, gender, IQ, years of education). Prior to regression analysis, collinearity was tested between the independent predictors entered into the statistical models in order to ensure that patterns of results were not biased by high amounts of collinearity among competing predictors.

As a further exploratory analysis, the total study sample was divided into two age categories (Younger adults [≤ 65; Range: 27–65 years] or older adults [> 65; Range: 66–82 years]) to explore further the differential associations between gait parameters, FC, neuropsychological performance and demographic factors within each age category, since 65 is commonly used as the age-cutoff to designate an older person in the community (American Institute for Research, 2015). Independent-samples *t*-tests were used to probe the differences in inter-network FC *z*-scores, cognitive performance scores, and gait parameters between both age category groups. Additionally, hierarchical linear stepwise regression analysis was performed within each age group to test the association between UW and DT gait parameters, inter-network FC *z*-scores, neuropsychological performance scores in the four domains and demographic variables (IQ, years of education). In the regression model, NW and DT spatiotemporal gait measures were entered as dependent variables; age and gender were entered as covariates in the first block; IQ, years of education and neuropsychological in 4 domains (6 variables), as well as the calculated inter-network FC levels (12 variables) were entered in the second block. The adopted significance threshold for all conducted statistical tests was *p* < 0.05 with Bonferroni-correction for multiple comparisons.

## Results

### Demographics, gait, and cognitive performance of the study participants

Table 1 displays demographic characteristics, neuropsychological, and gait performance scores in the overall study sample.

**TABLE 1:** Demographic, gait, and neuropsychological characteristics of the study group.

	Variable	
Demographics	Age (all group), *n* = 115	60.45 ± 13.75
	Gender	
	- Females, *n* = 64 (55.7%)	
	- Males, *n* = 51 (44.3%)	
	IQ	118.84 ± 8.13
	Years of education	16.19 ± 2.12
Neuropsychological performance (domain z-scores)	Processing speed	-0.08 ± 0.82
	Vocabulary	0.26 ± 0.82
	Episodic memory	-0.04 ± 0.97
	Fluid reasoning	-0.12 ± 0.73
Gait	UW velocity (m/s)	0.96 ± 0.18
	UW stride length (m)	1.2 ± 0.23
	UW stride regularity	0.76 ± 0.11
	UW step symmetry	0.81 ± 0.23
	UW step regularity	0.63 ± 0.16
	DT velocity (m/s)	0.84 ± 0.13
	DT stride length (m)	1.15 ± 0.16
	DT stride regularity (a.u)	0.61 ± 0.19
	DT step symmetry (a.u)	0.55 ± 0.19
	DT step regularity (a.u)	0.92 ± 0.34

Unless otherwise specified, all values are expressed as Mean ± SD. IQ, intelligence quotient; UW, usual walking; DT, dual-task; a.u, arbitrary unit.

### Association between gait, age and other demographic, neuropsychological performance, and inter-network functional connectivity measures in the overall study sample

**FIGURE 1:**
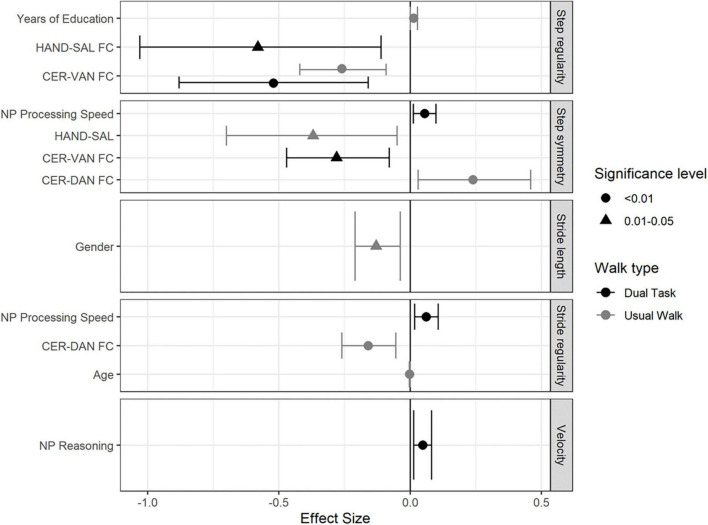
Forest plot demonstrating the independent predictors and effect sizes (95%CI) for the measured gait parameters in both walking conditions in the overall study group.

Figure 1 demonstrates the significant predictors and effect-sizes for gait performance parameters in both UW and DT conditions.

#### Usual walking

In the overall study group, CER-DAN FC and age were found to be negatively associated with stride regularity (β = −0.16; CI-95% [-0.26, -0.055]) and (β = −0.002; CI-95% [-0.003, 0]), respectively. Stride length was negatively associated with gender (β = −0.91; CI-95% [-0.13, -0.038]). CER-VAN FC and years of education were found to be significantly associated with step regularity (β = −0.62, CI-95% [-0.42, -0.92]) and (β = 0.14; CI-95% [0, 0.028]), respectively. Finally, step symmetry was negatively associated with HAND-SAL FC (β = −0.37, CI-95% [-0.7, -0.5], and CER-DAN FC (β = −0.91, CI-95% [0.03, −0.46]) (see Figure 1).

#### Dual-task

NP reasoning was positively associated with velocity (β = 0.048; CI-95% [0.014, 0.082]). Stride regularity was found to be positively associated with NP processing speed (β = 0.062; CI-95% [0.017, 0.11]). CER-VAN and HAND-SAL FC were both negatively associated with step regularity (β = −0.52, CI-95% [-0.88, -0.16]), and (β = −0.58; CI-95% [-1.03, -0.11]), respectively. Finally, step symmetry was found to be negatively associated with CER-VAN FC (β = −0.28, CI-95% [-0.47, -0.08], and positively NP processing speed (β = −0.055, CI-95% [0.013, -0.098]) (see Figure 1).

### Differences in gait, cognition, and inter-network functional connectivity between the two age groups

**FIGURE 2:**
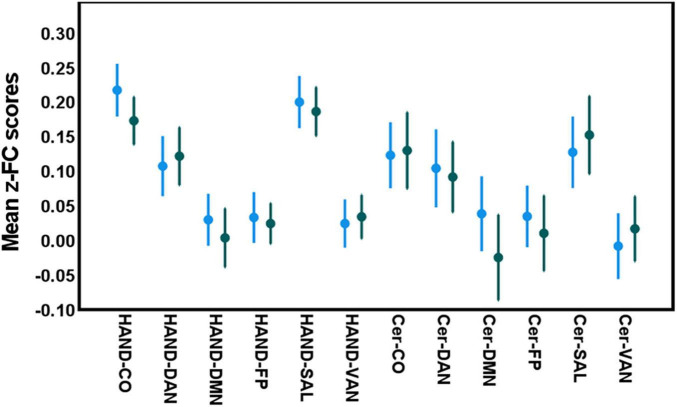
Between-group differences in inter-network connectivity levels in both age categories [*blue*: young adults (≤ 65 years); *green*: older adults age group (> 65 years). 2]. No significant differences were detected between both age groups in inter-network FC between these networks (Independent *t*-test; *p* > 0.05 all cases).

Significant differences were detected between the two age groups in processing speed, episodic memory, and fluid reasoning domain scores (two-sample *t*-test; *p* < 0.002, Bonferroni-corrected for all comparisons) (Table 2). No significant differences were detected in the spatio-temporal gait parameters in both walking conditions, as well as in the inter-network FC among the investigated networks between the two age groups (independent-samples *t*-test; *p* > 0.05 all cases; Figure 2).

**TABLE 2:** Demographic, gait, and neuropsychological characteristics as a function of age grouping.

	Variable	Younger adults (≤65), *n* = 60	Older adults (>65), *n* = 55	*P*-value
Demographics	Age	51.4 ± 13.36	70.27 ± 3.96	*p* < 0.001^§^ *
	Gender (F/M)	34 (56.7%)/26 (43.3%)	30 (54.5%)/25 (45.5%)	*p* = 0.22*^†^*
	IQ	118.83 ± 7.32	118.86 ± 8.99	*p* = 0.98^§^
	Years of education	16.12 ± 1.9	16.27 ± 23.34	*p* = 0.69^§^
Neuropsychological performance (domain z-scores)	Processing speed	0.16 ± 0.83	-0.34 ± 0.72	*p* < 0.001^§^ *
	Vocabulary	0.32 ± 0.72	0.2 ± 0.93	*p* = 0.44^§^
	Episodic memory	0.23 ± 0.92	-0.32 ± 0.95	*p* < 0.002^§^ *
	Fluid reasoning	0.13 ± 0.69	-0.38 ± 0.69	*p* < 0.001^§^ *
*Gait*	UW velocity (m/s)	0.96 ± 0.19	0.97 ± 0.18	*p* = 0.97^§^
	UW stride length (m)	1.19 ± 0.22	1.2 ± 0.24	*p* = 0.72^§^
	UW stride regularity (a.u)	0.78 ± 0.1	0.74 ± 0.11	*p* = 0.021^§¶^
	UW step symmetry (a.u)	0.79 ± 0.24	0.84 ± 0.22	*p* = 0.28^§^
	UW step regularity (a.u)	0.65 ± 0.16	0.61 ± 0.17	*p* = 0.27^§^
	DT velocity (m/s)	0.84 ± 0.13	0.84 ± 0.13	*p* = 0.76^§^
	DT stride length (m)	1.15 ± 0.13	1.15 ± 0.19	*p* = 0.95^§^
	DT stride regularity (a.u)	0.63 ± 0.2	0.59 ± 0.18	*p* = 0.22^§^
	DT step symmetry (a.u)	0.55 ± 0.2	0.54 ± 0.18	*p* = 0.62^§^
	DT step regularity (a.u)	0.88 ± 0.35	0.95 ± 0.34	*p* = 0.26^§^

Unless otherwise specified, all values are expressed as Mean ± SD. IQ, intelligence quotient; UW, usual walking; DT, dual-task; a.u, arbitrary units. ^†^Chi-square test. ^§^Independent-samples t-test, *p < 0.002 Bonferroni-corrected for multiple comparisons. ^¶^Result does not survive correction for multiple comparisons.

### Associations between gait, age and other demographic, neuropsychological performance, and inter-network functional connectivity measures in the different age groups

#### Younger adults group (≤ 65 years)

**FIGURE 3:**
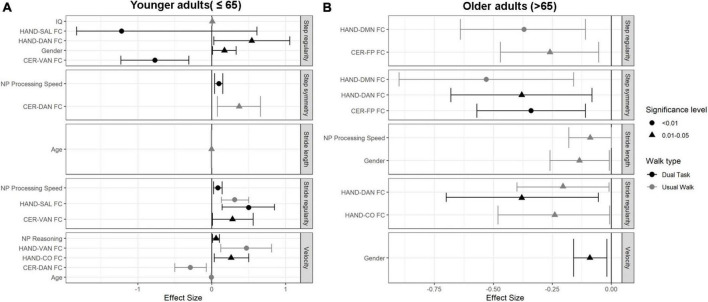
Forest plots demonstrating the independent predictors and effect sizes (95%CI) for the measured gait parameters in both walking conditions in **(A)** younger adults age group (≤65 years), and **(B)** older adults age group (>65 years).

The regression results are summarized in Figure 3A and Supplementary Table 1.

##### Usual walking

In this age group, velocity was associated negatively with Age, and CER-DAN FC (β = −0.096, CI-95% [0.39, 0.15]), and positively with HAND-VAN FC (β = 0.47, CI-95% [0.12, 0.81]. Stride length was negatively associated with age (β = −0.26, CI-95% [-0.008, 0]). HAND-SAL FC was positively associated with stride regularity (β = 0.31, CI-95% [0.13, 0.5]). Lastly, IQ was found to be positively associated with step regularity (β = 0.006, CI-95% [0, 0.012]), and CER-DAN FC was found to be positively associated with step symmetry (β = 0.37, CI-95% [0.076, 0.66]).

##### Dual-task walking

Fluid reasoning and HAND-CO FC were positively associated with velocity (β = 0.057, CI-95% [0.011, 0.10]; β = 0.26, CI-95% [0.033, 0.49], respectively). Stride regularity was found to be positively associated with processing speed (β = 0.37, CI-95% [0.025, 0.14]), HAND-SAL (β = 0.49, CI-95% [0.14, 0.85]), and CER-VAN FC (β = 0.28, CI-95% [0.008, 0.56]). Step regularity was negatively associated with CER-VAN FC (β = −0.77, CI-95% [-1.23, -0.31]), and HAND-SAL FC (β = −1.22, CI-95% [-1.83, -0.61]), as well as positively associated with HAND-DAN FC (β = 0.54, CI-95% [0.028, 1.056]), and female gender (β = 0.17, CI-95% [0.028, 1.056]). Finally, processing speed was found to be significantly associated with step symmetry (β = 0.096, CI-95% [0.39, 0.15]) (see Figure 3A and Supplementary Table 1).

#### Older adults age group (>65 years)

The regression results for this age group are summarized in Figure 3B and Supplementary Table 2.

##### Usual walking

Negative associations were found between stride length, male gender and processing speed (β = −0.13, CI-95% [-2.26, -0.009]); (β = −0.09, CI-95% [-0.18, 0]), respectively. HAND-DAN FC (β = −0.2, CI-95% [-0.4, -0.1]), and HAND-CO FC (β = −2.4, CI-95% [-0.48, -0.007]) were found to be negatively associated with stride regularity. Step regularity was negatively associated with HAND-DMN (β = −0.37, CI-95% [-0.64, -0.11]) and CER-FP FC (β = −0.26, CI-95% [-0.47, -0.05]). Finally, HAND-DMN FC (β = −0.53, CI-95% [-0.9, -0.16]) was found to be negatively associated with step symmetry.

##### Dual-task walking

Gender was found to be associated with velocity, such that male gender was associated with faster velocity (β = −0.09, CI-95% [-0.16, -0.2]). Stride regularity was negatively associated with HAND-DAN FC (β = −0.38, CI-95% [-0.71, -0.05]). Finally, step symmetry was negatively associated with CER-FP and HAND-DAN FC (β = −0.34, CI-95% [-0.57, -0.11]); (β = −0.38, CI-95% [-0.68, -0.08]), respectively (Figure 3B).

## Discussion

We evaluated the association between cognitive performance, inter-network FC, and gait performance in UW and DT walking conditions both as a function of aging, and, in secondary analyses, in two age sub-groups; people below and above 65 years of age. We found that across the whole study sample, UW was associated with FC between motor and sustained attention networks, while DT gait performance was associated with inter-network FC between motor and divided attention networks as well as processing speed. When investigating these associations in younger and older age groups, we observed that UW was associated with inter-network FC between motor and sustained attention networks. On the other hand, DT performance was associated with cognitive performance, as well as inter-network connectivity between motor and divided attention networks (VAN and SAL). Finally, regressions conducted in the older age group (> 65 years of age) showed that increased integration between motor, dorsal, and ventral attention, as well as default-mode networks, was negatively associated with UW gait performance.

In the overall study sample, inter-network FC among motor control regions (cerebellar and motor cortex) and attention-related (salience, dorsal-attention, and ventral attention) networks, in addition to age and gender, were significantly associated with stride length, stride regularity, step length, and step symmetry in UW conditions. Corroborating previous reports, this finding emphasizes the role of sustained “top-down” attention allocation, which enables filtering and the suppression of external stimuli and distractors during walking (Yogev-Seligmann et al., 2008; Ferrazzoli et al., 2017; Maidan et al., 2019). In fact, a relationship between reduced FC within the DAN network and poorer gait performance was observed in Parkinson’s disease (PD) individuals with freezing of gait (FOG) in comparison to PD patients without FOG and to healthy age-matched controls (Maidan et al., 2019). In DT conditions, the performance in the same gait parameters was associated with NP reasoning and processing speed, as well as CER-VAN, and HAND-SAL inter-network FC. The interplay between motor and ventral-frontoparietal attention networks is implicated in eliciting “bottom-up” external attention and may play a role in detecting unattended or unexpected stimuli and triggering shifts of attention to the cognitive task in DT walking condition (Vossel et al., 2014; Yuan et al., 2015).

The observed findings in the younger adults group show that cognitive abilities play a role in the maintenance of gait performance in the context of aging and the face of disease (Bettcher et al., 2019). Namely, UW gait performance parameters were associated with IQ, as well as inter-network FC between motor and attention networks (i.e., CER-DAN, HAND-SAL, HAND-VAN). During DT, this age group showed reliance on additional neural and cognitive resources including fluid reasoning and processing speed domain scores, as well as internetwork FC between motor and divided attentional networks such as the CER-VAN and HAND-CO. This pattern of inter-network integration between motor, cingulo-opercular, and salience networks at rest is believed to enable efficient switching between several functional brain networks. This can facilitate access to attention and working memory resources due to the rising demands of the DT walking condition (Menon and Uddin, 2010; Vaden et al., 2013), enable sustained alertness, and elicit a faster response (Coste and Kleinschmidt, 2016).

Interestingly, distinct inter-network FC patterns were further observed to be associated with the different gait spatiotemporal measures. For example, while stride regularity was associated with FC integration between HAND-SAL and CER-VAN networks, step regularity was additionally associated with HAND-DAN FC. Though both step regularity and stride regularity gait measures are related, these represent different biomechanical components. Step regularity reflects unilateral measures of coordination and smoothness, yet, stride regularity reflects the bilateral duality of the closed kinematic network, also including the inter-limb coordination and balance support (James et al., 2020). In line with this, step regularity showed higher reliance on motor-SAL and motor-DAN FC integration in DT specifically indicating a more automated motor scheme with the reliance on attentional resources for the dual task. Stride regularity, on the other hand, relied on the integration between motor and SAL network, in addition to cerebellum and VAN networks that are involved more in balance, coordination and initiation of movements. Together, these findings further highlight the role of high-order cognitive functions in sustained gait performance in cognitively demanding DT conditions in younger adults.

On the other hand, different patterns were observed in the older age group. The decline in overall cognitive reserve capacities in the older adult group (> 65), as reflected by the NP domains scores, may have led to distinct patterns of inter-network FC associated with UW and DT gait performance, compared to the younger age group. Specifically, this age group demonstrated comparable, yet negative, inter-network FC and UW gait measures associations to those of the younger adults group in the DT condition. Moreover, in DT, this older age group demonstrated associations between inter-network FC between sustained attention and motor networks (HAND-DAN, CER-FP) only. These results suggest that with increased age, and a decline in cognitive functions, additional neural resources are required for executing relatively basic walking tasks (Glisky, 2007; Holtzer et al., 2012; Martin et al., 2013; Poole et al., 2019). In line with this, our obtained findings in this age group revealed that due to the reduced cognitive reserve, older individuals rely on additional neural resources to compensate for and maintain preserved gait performance during UW tasks. As a result, FC integration between HAND-DMN and HAND-CO was associated with UW parameters in addition. Such patterns might be reflective of the increased neural capacities required for carrying out routine activities with aging. Inter-network integration between motor and DMN networks was previously reported to be associated with poorer gait performance in older adults with mild cognitive impairments. The sub-optimal suppression of the DMN might be indicative of attentional impairments, resulting in reduced sustained attentional capacities and increased sensitivity to distraction (Crockett et al., 2017; Lo et al., 2021). Furthermore, DT gait parameters (i.e., stride regularity and step symmetry) were negatively associated with FC between CER-FP and HAND-DAN networks in the older age group. Inter-network integration between motor and executive control brain networks was reported to be associated with faster walking, especially during the DT condition. Moreover, declined executive functioning was linked with poorer dual-tasking (Ohsugi et al., 2013), slower gait speed (Cohen et al., 2016), and increased risk of falls in older adults (Mirelman et al., 2012). Interestingly, this age group showed no reliance on divided attention networks during the cognitively demanding walking condition. This suggests that older participants allocated more resources to the walking condition at the expense of the cognitive task (Li et al., 2018). These differential relationships as a function of age group are reflective of the higher utilization cognitive and neural resources. With time, these compensatory processes may not suffice to counteract the ongoing changes to the nervous system, potentially leading to more pronounced gait dysfunctions and perhaps to falls.

While the results of this study align with previous findings, the results based on the analysis within the different age groups demonstrate that the decline of cognitive reserve with aging leads to alterations and re-configuration of the neural mechanisms underlying gait performance, reflecting the dynamic utilization of these resources throughout the life span. Nevertheless, the limitations of the present study should be addressed. Firstly, this was a cross-sectional analysis, investigating the integration dynamics among large-scale brain networks, cognition, and gait performance throughout the age-span. Hence, we can only infer about the relationships among gait performance, cognitive capacities, and inter-network FC patterns. Longitudinal study designs would allow further examining these dynamics in depth, and potentially establish the underlying causal relationships among these neural and behavioral domains. Additionally, enrolling larger numbers of participants in decadic age brackets would enable a more accurate depiction of the temporal dynamics among these factors. Another limitation of the present study is that the networks modeled here were based on a predefined network atlas and not based on individualized network parcellations. While this may result in networks that do not precisely reflect network organization for each participant, the parcellation was previously found to be consistent across participants from different age groups, and utilizing this external network scheme facilitates comparisons between participants by ensuring network consistency among all participants across a wide range of ages. Finally, while we were primarily interested in the effects of the cognitive load on gait performance, the participants’ level of engagement was not assessed, a factor which might affect FC within involved brain networks as performance in both walking tasks.

Based on these findings, we suggest that the ability to deploy higher cognitive and neural resources to support gait appears to be dynamic across the aging spectrum and may be employed as a compensatory mechanism to minimize performance deficits in older adults. Future studies should build on the present work and assess whether changes in gait can also be used to predict underlying incident connectivity alterations and cognitive pathology, and examine whether these may serve as useful biomarkers for the early detection of neurological deficits and other adverse health outcomes.

## Data availability statement

The data supporting the findings reported here will be available upon reasonable request by researchers who meet the criteria for access to confidential data.

## Ethics statement

This study was approved by the Local Columbia University Medical Center Institutional Review Board. The patients/participants provided their written informed consent to participate in this study.

## Author contributions

AD, JH, AM, and IM conceptualized the study design, analyzed the data, and outlined the manuscript. EV, CH, and YS collected, analyzed the data, and revised the manuscript. All authors contributed to the article and approved the submitted version.
