# Supplementary material for: Effects of aging on cognitive and brain inter-network integration patterns underlying usual and dual-task gait performance
Source: Front Aging Neurosci. 2022 Sep 28;14:956744. doi: 10.3389/fnagi.2022.956744 (PMC9557358; doi:10.3389/fnagi.2022.956744)
Supplement: Supplementary file 1 [file Table_1.docx]

| **Dependent variables** | **Model** | | | | | **Independent predictors** | | |
| --- | --- | --- | --- | --- | --- | --- | --- | --- |
|  | **R^2^** | **Adjusted R^2^** | **F** | ***df*** | **P- value** | **Β** | **t-value** | **P-value** |
| Usual walking | | | | | | | | |
| UW velocity | 0.28 | 0.24 | 6.87 | 1-57 | <0.001 |  |  |  |
| - Age |  |  |  |  |  | -0.006 | -3.44 | 0.001 |
| - HAND-VAN FC |  |  |  |  |  | 0.47 | 2.7 | 0.009 |
| - CER-DAN FC |  |  |  |  |  | -0.29 | -2.7 | 0.009 |
| UW stride length | 0.068 | 0.051 | 4.06 | 1-57 | 0.049 |  |  |  |
| - Age |  |  |  |  |  | -0.004 | -2.02 | 0.049 |
| UW stride regularity | 0.41 | 0.15 | 11.08 | 1-57 | 0.002 |  |  |  |
| - HAND-SAL FC |  |  |  |  |  | 0.31 | 3.33 | 0.002 |
| UW step regularity | 0.076 | 0.06 | 4.62 | 1-57 | 0.036 |  |  |  |
| - IQ |  |  |  |  |  | 0.006 | 2.15 | 0.036 |
| UW step symmetry | 0.10 | 0.086 | 6.36 | 1-57 | 0.015 |  |  |  |
| - CER-DAN FC |  |  |  |  |  | 0.37 | 2.52 | 0.015 |
| Dual-task walking | | | | | | | | |
| DT velocity | 0.20 | 0.17 | 6.82 | 1-57 | 0.002 |  |  |  |
| - Fluid reasoning |  |  |  |  |  | 0.06 | 3.46 | 0.017 |
| - HAND-CO FC |  |  |  |  |  | 0.26 | 2.29 | 0.026 |
| DT stride regularity | 0.27 | 0.23 | 6.67 | 1-57 | <0.001 |  |  |  |
| - Processing speed |  |  |  |  |  | 0.083 | 2.88 | 0.006 |
| - HAND-SAL FC |  |  |  |  |  | 0.49 | 2.79 | 0.007 |
| - CER-VAN FC |  |  |  |  |  | 0.28 | 0.06 | 0.044 |
| DT step regularity | 0.39 | 0.34 | 8.32 | 1-57 | <0.001 |  |  |  |
| - CER-VAN FC |  |  |  |  |  | -0.77 | -3.38 | 0.001 |
| - HAND-SAL FC |  |  |  |  |  | -1.22 | -4.04 | <0.001 |
| - Gender |  |  |  |  |  | 0.17 | 3.15 | 0.036 |
| - HAND-DAN FC |  |  |  |  |  | 0.54 | 2.11 | 0.04 |
| DT step symmetry | 0.17 | 0.15 | 11.24 | 1-57 | 0.001 |  |  |  |
| - Processing speed |  |  |  |  |  | 0.96 | 3.35 | 0.001 |

**Supplementary Table 1:** Results of the step-wise regression analysis in the younger adults group (≤65 years).

*Abbreviations: IQ=intelligence quotient, UW= usual walking, DT= dual-task*. Spatio-temporal gait measures were entered as a dependent variable, age and gender were entered as covariates in the first block, and neuropsychological (6 variables) and the calculated inter-network FC levels (12 variables) were entered in the second block of the regression model.
