# Supplementary material for: Effects of aging on cognitive and brain inter-network integration patterns underlying usual and dual-task gait performance
Source: Front Aging Neurosci. 2022 Sep 28;14:956744. doi: 10.3389/fnagi.2022.956744 (PMC9557358; doi:10.3389/fnagi.2022.956744)
Supplement: Supplementary file 2 [file Table_2.docx]

**Supplementary Table 2:** Results of the step-wise regression analysis in the older adult group (>65 years).

| **Dependent variables** | **Model** | | | | | **Independent predictors** | | |
| --- | --- | --- | --- | --- | --- | --- | --- | --- |
|  | **R^2^** | **Adjusted R^2^** | **F** | ***df*** | **P- value** | **Β** | **t-value** | **P-value** |
| Usual walking | | | | | | | | |
| UW stride length | 0.15 | 0.12 | 4.42 | 1-51 | 0.017 |  |  |  |
| - Gender |  |  |  |  |  | -0.13 | -2.16 | 0.036 |
| - Processing speed |  |  |  |  |  | -0.09 | -2.02 | 0.049 |
| UW stride regularity | 0.2 | 0.17 | 6.19 | 1-51 | 0.004 |  |  |  |
| - HAND-DAN FC |  |  |  |  |  | -0.2 | -2.12 | 0.039 |
| - HAND-CO FC |  |  |  |  |  | -0.24 | -2.07 | 0.043 |
| UW step regularity | 0.29 | 0.23 | 7.24 | 1-51 | 0.002 |  |  |  |
| - HAND-DMN FC |  |  |  |  |  | -0.37 | -2.86 | 0.007 |
| - CER-FP FC |  |  |  |  |  | -0.26 | -2.53 | 0.015 |
| UW step symmetry | 0.15 | 0.13 | 8.53 | 1-51 | 0.005 |  |  |  |
| - HAND-DMN FC |  |  |  |  |  | -0.53 | -2.92 | 0.005 |
| Dual-task walking | | | | | | | | |
| DT velocity | 0.12 | 0.098 | 6.46 | 1-51 | 0.014 |  |  |  |
| - Gender |  |  |  |  |  | -0.091 | -2.54 | 0.014 |
| DT stride regularity | 0.10 | 0.083 | 5.51 | 1-51 | 0.023 |  |  |  |
| - HAND-DAN FC |  |  |  |  |  | -0.38 | -2.35 | 0.023 |
| DT step symmetry | 0.27 | 0.24 | 9.11 | 10.5 | 0.002 |  |  |  |
| - CER-FP FC |  |  |  |  |  | -0.34 | -2.96 | 0.005 |
| - HAND-DAN FC |  |  |  |  |  | -0.38 | -2.56 | 0.014 |

*Abbreviations: IQ=intelligence quotient, UW= usual walking, DT= dual-task.* Spatio-temporal gait measures were entered as a dependent variable, age and gender were entered as covariates in the first block, and neuropsychological (6 variables) and the calculated inter-network FC levels (12 variables) were entered in the second block of the regression model.
